# *Bacteroides thetaiotaomicron* and *Faecalibacterium prausnitzii* served as key components of fecal microbiota transplantation to alleviate colitis

**DOI:** 10.1152/ajpgi.00303.2023

**Published:** 2024-03-19

**Authors:** Binqiang Xu, Yang Fu, Nuoming Yin, Wenfei Qin, Zehua Huang, Wei Xiao, Huizhen Huang, Qixiang Mei, Junjie Fan, Yue Zeng, Chunlan Huang

**Affiliations:** ^1^Shanghai Key Laboratory of Pancreatic Diseases, Shanghai JiaoTong University School of Medicine, Shanghai, People’s Republic of China; ^2^Department of Gastroenterology, Shanghai General Hospital, Shanghai JiaoTong University School of Medicine, Shanghai, People’s Republic of China; ^3^School of Health Science and Engineering, University of Shanghai for Science and Technology, Shanghai, People’s Republic of China; ^4^Shanghai General Hospital of Nanjing Medical University, Shanghai, People’s Republic of China

**Keywords:** Bacteroides thetaiotaomicron, Faecalibacterium prausnitzii, fecal microbiota transplantation, gut microbiota, inflammatory bowel disease

## Abstract

Fecal microbiota transplantation (FMT) is a promising therapy for inflammatory bowel disease (IBD) via rectifying gut microbiota. The aim of this study was to identify a mechanism of how specific bacteria-associated immune response contributes to alleviated colitis. Forty donors were divided into high (*donor H*) and low (*donor L*) groups according to the diversity and the abundance of *Bacteroides* and *Faecalibacterium* by 16S rRNA sequencing. FMT was performed on dextran sulfate sodium (DSS)-induced colitis in mice. Mice with colitis showed significant improvement in intestinal injury and immune imbalance after FMT with *group donor H* (*P* < 0.05). *Bacteroides thetaiotaomicron* and *Faecalibacterium prausnitzii* were identified as targeted strains in donor feces by real-time PCR and droplet digital PCR. Mice with colitis were treated with mono- or dual-bacterial gavage therapy. Dual-bacterial therapy significantly ameliorated intestinal injury compared with mono-bacterial therapy (*P* < 0.05). Dual-bacterial therapy increased the M2/M1 macrophage polarization and improved the Th17/Treg imbalance and elevated IL-10 production by Tregs compared with the DSS group (*P* < 0.05). Metabolomics showed increased abundance of lecithin in the glycerophospholipid metabolism pathway. In conclusion, *B. thetaiotaomicron and F. prausnitzii*, as the key bacteria in donor feces, alleviate colitis in mice. The mechanism may involve increasing lecithin and regulating IL-10 production of intestinal Tregs.

**NEW & NOTEWORTHY** We demonstrate that donors with high abundance of *Bacteroides* and *Faecalibacterium* ameliorate dextran sulfate sodium (DSS)-induced colitis in mice by fecal microbiota transplantation (FMT). The combination therapy of *Bacteroides thetaiotaomicron* and *Faecalibacterium prausnitzii* is superior to mono-bacterial therapy in ameliorating colitis in mice, of which mechanism may involve promoting lecithin and inducing IL-10 production of intestinal Tregs.

## Graphical abstract

**Figure:**
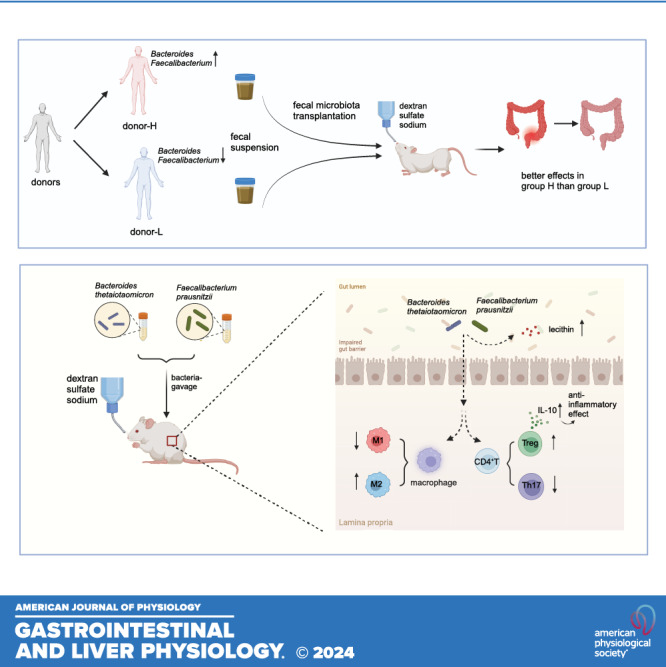

## INTRODUCTION

Inflammatory bowel disease (IBD) is a chronic nonspecific inflammatory disease in gut, including ulcerative colitis (UC) and Crohn’s disease (CD). In recent years, its prevalence and incidence have been increasing, bringing a greater global burden to society (1). The pathogenesis of IBD is an aberrant immune response by a genetically susceptible host against the gut microbiota triggered by environmental factors (2, 3).

The pathogenesis of UC is associated with the gut dysbiosis, particularly microbiota shifts belonging to *Bacteroides* and *Faecalibacterium* (4). *Bacteroides*, the major microbial genus of Bacteroidetes, are known to decrease in patients with IBD, even lower in active phase than in remission (5–7). Some species of *Bacteroides*, such as *B. thetaiotaomicron and B. ovatus*, have exhibited beneficial anti-inflammatory functions (8–10). *B. thetaiotaomicron* was found to ameliorate the severity of colitis model in dextran sodium sulfate (DSS) and interleukin (IL)-10 knockout models of colitis (11). Besides, *Faecalibacterium* is another dominant genus, which is significantly decreased in IBD (7). The decrease of *F. prausnitzii* and its metabolite butyrate affected the intestinal homeostasis in patients with active IBD, while its supplementation could ameliorate DSS-induced colitis by maintaining T helper (Th) 17 cell and regulatory T-cell (Treg) balance and inhibiting *Candida albicans* colonization (12–16). *F. prausnitzii* can also be used as a biomarker for assessing disease activity, treatment efficacy, and recurrence in IBD (6, 17).

Fecal microbial composition of donors is considered a crucial factor in enhancing FMT efficacy by modifying dysbiosis (18–20). A nonrandomized open-label study demonstrated that donor feces with higher abundance of specific bacteria exhibited higher remission rates in patients with UC receiving FMT therapy, indicating a significant impact of different gut microbiota composition of donors on FMT efficacy, of which the exact mechanism was unknown (21).

The incidence and severity of IBD are closely related to the immune activation to corresponding microbiota (22). *B. thetaiotaomicron* could improve colitis by activating aryl hydrocarbon receptor (AHR) to regulate the differentiation of Tregs and T helper cells (9). *B. fragile* could promote the production of IL-10 in regulatory CD4^+^ T cells upon binding with B cells (23). *F. prausnitzii* intervention could decrease Th17 differentiation and attenuate colitis through inhibiting histone deacetylase 3 (HDAC3) and c-Myc-related metabolism in T cells (13).

In our study, the gut microbiota of donors was screened by 16S rRNA sequencing, and the efficacy of *Bacteroides* and *Faecalibacterium* in donors on FMT in DSS-induced colitis in mice was investigated. Targeted species in these two genera were identified and then applied in mice with colitis to explore the mechanism of how specific bacteria-associated immune response contributes to alleviated colitis.

## MATERIALS AND METHODS

### Animals

Male C57BL/6 mice (6–8 wk of age, 20–22 g) were obtained from Shanghai SLAC Laboratory Animal Co. Ltd. (PR China). Mice were housed under specific pathogen-free (SPF) conditions with a room temperature of 24 ± 2°C and a 12-h light/dark cycle. The scheme of all animal experiments was approved by the experimental Animal Ethical Committee of Shanghai General Hospital (2020AW095), affiliated with Shanghai JiaoTong University School of Medicine.

### Experimental Design

In our study, 40 healthy volunteers were enrolled as donors according to the scheme shown in Supplemental Fig. S1. The gut microbiota of donors was screened by 16S rRNA sequencing. We then ranked the donors in descending order based on the abundance of *Bacteroides* and *Faecalibacterium* and microbial diversity. The ranking top 20 donors were designated as *group donor H* and the remaining 20 donors were designated as *group donor L* (Supplemental Fig. S1). The study involving samples from volunteers was reviewed and approved by the Ethics Committee of Shanghai General Hospital (No. 2021035). All volunteers provided written consent.

To observe the impact of different donors on the efficacy of FMT, mice were randomly divided into eight groups (*n* = 6–8): a control (CON) group, a DSS-induced colitis (DSS) group, and the DSS+FMT (H1, H2, H3, L1, L2, and L3) groups.

Colitis model in mice was induced by administering 2.5% (wt/vol) DSS (MW = 36,000–50,000, MP Biomedicals, CA) in drinking water for 7 days.

Donors were requested to collect the stool in a container and deliver it to the hospital within 6 h after defecation in the morning. Before being processed, stool samples were stored in a biosafety cabinet at ambient temperature (20–30°C) (24). The processing of FMT suspension was conducted within 2 h after collection. A stool sample of 10 g was diluted with 50 mL of sterile 0.9% saline and then vortexed for 10 min. The mixture was filtered twice with sterile gauze to remove the impurities. The filtrate was mixed with glycerol (10%) then divided into small portions and frozen at −80°C in centrifuge tubes for later FMT processing. On the day of fecal infusion, fecal suspension was thawed in a warm water bath (37°C) and infused within 6 h from being thawed. After modeling colitis, mice in the FMT group were treated with FMT (1 mL/g body wt/day) by gavage for 5 days, whereas mice in the CON group and the DSS group were treated with PBS instead.

In our bacteria-gavage therapy experiments, mice were randomly divided into four groups (*n* = 6–13): CON group, DSS group, DSS+*Bacteroides thetaiotaomicron*-treated (DSS + Bt) group, DSS+*Faecalibacterium prausnitzii*-treated (DSS+Fp) group, and DSS+*Bacteroides thetaiotaomicron*+*Faecalibacterium prausnitzii*-treated (DSS+ Bt+Fp) group.

For the mono-bacterial therapy, mice were administered with 0.2 mL of *B. thetaiotaomicron* suspension in DSS+Bt group or *F. prausnitzii* in the DSS+Fp group [1 × 10^9^ colony-forming units (CFU)/mL] by oral gavage daily for 7 days. As for the DSS+Bt+Fp group, mice were administered with 0.2 mL of mixed dual-bacterial solution with *B. thetaiotaomicron* and *F. prausnitzii* (1 × 10^9^ CFU/mL) in 1:1 ratio. The DSS group was administered 0.2 mL of PBS solution.

### Histological Analysis

After the mice were euthanized, fresh colon tissues were fixed in 4% paraformaldehyde at 4°C overnight and stained with hematoxylin and eosin (H&E; Servicebio, Wuhan, PR China). Morphological changes were examined by light microscopy (Leica, Germany) at a magnification of ×200 or 400. The histopathological changes of the colon were evaluated according to the scoring criteria (25).

### Immunofluorescence

Colon tissue sections were heated at 60°C for 1 h and then put into Leica Autostainer XL (Leica) for hydration in certain steps (xylene for 40 min, 100% ethanol for 10 min, 95% ethanol for 10 min, 80% ethanol for 5 min, 70% ethanol for 5 min, and doubly distilled water for 3 min). Antigen retrieval was performed with sodium citrate solution (Sangon Biotech, Shanghai, PR China). After repeated washing in PBS, slides were blocked with immunostaining blocking buffer (Sangon Biotech, Shanghai, PR China) for 1 h at room temperature and incubated with primary antibodies against Claudin-1 (ab211737, Abcam, Cambridge, MA) and ZO-1 (13663, Cell Signaling Technology, Boston, MA), which were diluted with primary antibody dilution buffer (Sangon Biotech, Shanghai, PR China) at 4°C overnight. Slides were washed with PBS and incubated with Alexa Fluor 488 AffiniPure Donkey Anti-Rabbit Antibody (Yeason, Shanghai, PR China) at room temperature for 1 h. The slides were then stained with dihydrochloride (DAPI) (Yeason, Shanghai, PR China) for 5 min to visualize the nuclei.

### Real-Time PCR

Total RNA was extracted from approximately 50 mg colon tissue using TRIzol reagent (Accurate Biology, Hunan, PR China), according to the manufacturer’s protocol. Complementary DNA synthesis was performed using HyperScript III RT SuperMix (EnzyArtisan, Shanghai, PR China) for quantitative PCR with genomic DNA remover. Bacterial DNA was extracted from stool samples with an E.Z.N.A. stool DNA kit (Omega, Norcross, GA). Real-time PCR was performed using the SYBR Green reagents (EnzyArtisan, Shanghai, PR China) on QuantStudio 6 Flex real-time PCR systems (Thermo Scientific, Waltham, MA) according to a certain reaction (program predenaturation, 95°C, 30 s, 1 cycle; denaturation, 95°C, 10 s, 40 cycles; annealing and extension, 60°C, 30 s, 40 cycles). Gene expression was measured by the 2^−ΔΔCT^ method. The primer sequences used are listed in the Supplemental Table S1.

### Droplet Digital PCR

Stool DNA was extracted using a DNA extraction kit, and primer probes were added to configure the reaction system, with the probe sequences shown in Supplemental Table S1. PCR reaction solution (20 μL) from the above system was transferred to sample wells of the DG8 cartridge, and 70 μL of droplet generation oil were added to the oil wells. The reaction droplets were prepared by using the QX200TM droplet digital PCR (ddPCR) Instrument’s droplet generator. The PCR amplification in this experiment was performed on the Bio-Rad T100 PCR instrument and was carried out according to a certain reaction program (enzyme activation, 95°C, 10 min, 1 cycle; denaturation, 94°C, 30 s, 40 cycles; annealing/extension 60°C, 1 min, 40 cycles; hold 98°C, 10 min, 1 cycle). Finally, the fluorescence signal of each droplet was detected by the QX200 Droplet Reader. The QuantaSoft then automatically processed the data to obtain the copy number concentration of the target sequence in the PCR reaction system (unit: copies/μL).

### Bacterial Culture

The bacterial strains *B. thetaiotaomicron* (ATCC 29148) and *F. prausnitzii* (ATCC 27766) were grown at 37°C in an anaerobic incubator containing 80% N_2_, 10% CO_2_, and 10% H_2_. Bacteria were cultured in LYHBHI medium containing brain-heart infusion (37 g/L, BD Bioscience, San Jose, CA), yeast extract (5 g/L, Oxoid, Basingstoke, UK), hemin (5 mg/L, Macklin, Shanghai, PR China), supplied with cellobiose (1 g/L, Macklin, Shanghai, PR China), maltose (1 g/L, Macklin, Shanghai, PR China), and cysteine (0.5 g/L, Macklin, Shanghai, PR China). After being cultured for 24 h, the bacteria were harvested by centrifugation (1,500 rpm for 15 min), washed three times with PBS, and finally resuspended in PBS to a concentration of approximately 10^9^ CFU/mL.

### Lamina Propria Mononuclear Cells Isolation

Lamina propria mononuclear cells (LPMCs) were isolated from freshly obtained colon tissue as previously described (26). Briefly, after the mice were euthanized, the colon was isolated and placed in ice-cold PBS. The feces in the intestinal lumen were cleared, and then the enteric cavity was opened longitudinally, cut into 1-cm pieces, and washed in ice-cold PBS. The intestinal pieces were placed in 5 mL of predigestion solution [1 × Hank’s balanced salt solution (HBSS, Servicebio, Wuhan, PR China) containing 5 mM ethylene diamine tetraacetic acid (EDTA, Sangon Biotech, Shanghai, PR China) and 1 mM dithiothreitol (DTT, Yeason, Shanghai, PR China)] for 20 min at 37°C under slow rotation (40 *g*) in a thermal incubator in a 50-mL tube. The remaining pieces were passed through a 100-μm cell filter. Repeat the preceding steps again. The remaining tissue was cut into 1-mm pieces with scissors and collected into 50-mL tubes containing 5 mL of digestion solution. The digestion solution was prepared by dissolving 0.05 g of collagenase D (Roche, Basel, Switzerland), 0.05 g of DNase I (Sigma, St. Louis, MO), and 0.3 g of dispase II (Roche, Basel, Switzerland) in 100 mL of 1× PBS. Pieces were digested by incubating for 30 min at 37°C under slow rotation (40 *g*). After incubation, the cell solution was strongly vortexed for 20 s and passed through a 40-μm cell filter set on a 50-mL tube. The filtrate was placed in a fresh 50-mL tube and centrifuged for 10 min at 20°C, 500 *g*. The supernatant was removed, and the precipitate was resuspended with 5 mL of cold fluorescence-activated cell sorting (FACS) [3% (vol/vol) FCS in 1× PBS] and pipetted into a 15-mL Eppendorf (EP) tube.

### Cell Culture

For cytokine analysis, cells were cultured in a concentration of 5 × 10^5^ cells/well in 96 well microtiter plates (Corning, MA) with 200 μL of medium at 37°C in a humidified atmosphere with 5% CO_2_. The culture medium was RPMI-1640 medium (Bioagrio, Shanghai, PR China) containing 10% FCS (Bioagrio, Shanghai, PR China) and 1% penicillin and streptomycin. Leukocyte activation cocktail, with BD GolgiPlug (BD PharMingen, San Diego, CA) at 2 μL/mL was added 4 h before the end of culture.

### Flow Cytometry

Flow cytometric analysis was performed on a LSR Fortessa (BD Biosciences, San Jose, CA) instrument and FlowJo software. Dead cells were excluded by staining with Fixable Viability Stain 510 (BD PharMingen, San Diego, CA). LPMCs were stained with the following markers: CD45-APC-CY7, CD11b-FITC, F4/80-BV605, CD86-BV421, CD3-FITC, CD4-BB700, CD25-BV421, LY-6C-PE-CY7, CD11c-PE-CY7, and I-A/I-E-BB700 (BD PharMingen, San Diego, CA). After surface antigens were stained, the cells were then fixed and permeabilized by Transcription Factor Buffer Set (BD PharMingen, San Diego, CA). Then, cells were stained for intracellular markers: CD206-PE, Foxp3-PE, IL-10-APC, and IL-17A-Brilliant Violet711 (Biolegend, San Diego, CA). The gating strategy is shown in Supplemental Fig. S2.

### 16S rRNA Sequencing

Genomic DNA was extracted from the contents of the ileocecum using the E.Z.N.A. stool DNA kit (Omega, Norcross, GA) according to the manufacturer’s instructions and amplified using forward primer 5′-TACGGRAGGCAGCAG-3′ and reverse primer 5′- AGGGTATCTAATCCT-3′. 16S rRNA high-throughput sequencing was performed on an Illumina HiSeq platform (Illumina, San Diego, CA) according to the standard protocols by Majorbio Bio-Pharm Technology. The raw sequencing reads of this study are openly available in BioProject at http://www.ncbi.nlm.nih.gov/bioproject/1070265, reference number PRJNA1070265.

### Microbiome Analysis

The sequences were filtered with fastp (0.19.6) and merged with FLASH (v1.2.11). The high-quality sequences were denoised using the DADA2 plugin in QIIME2 (v. 2020.2) with recommended parameters, resulting in the generation of sequence variants known as amplicon sequence variants (ASVs). The naive Bayes consensus taxonomy classifier implemented in QIIME2 and SILVA 16S rRNA database (v138) was used for taxonomic assignment of ASVs. Analysis of the gut microbiota was carried out using the Majorbio Cloud platform (https://cloud.majorbio.com). Alpha diversity indices, including the Shannon index and the Simpson index, were calculated with Mothur v1.30.1. Principal coordinate analysis (PCoA) based on Bray–Curtis dissimilarity using the Vegan v2.5-3 package was performed to analyze the microbial communities in different samples.

### Metabolomics Analysis

The cecal contents were collected, and the fecal metabolites were extracted with 400 μL of extraction solution (methanol: water = 4:1 vol:vol) containing 0.02 mg/mL of the internal standard (l-2-chlorophenylalanine) per 100 mg of sample. Five replicates were set up for each group. To determine the stability of the analysis, an aliquot of mixed quality control (QC) samples was also set up. The above mixtures were crushed, ultrasonicated, and centrifuged, and the supernatants were transferred to sample vials for LC-MS/MS analysis. The LC-MS raw data were imported into the metabolomics processing software Progenesis QI (Waters Corporation, Milford, MA) for processing. The data matrix after library search was uploaded to the online platform of majorbio cloud platform (cloud.majorbio.com) for analysis.

### Statistics Analysis

All the measured data were displayed as means ± SE, and the analysis was performed using GraphPad prism 9.0 software. Comparisons between two groups that follow a normal distribution are conducted using the Student’s *t* test. For comparisons involving more than two groups, one-way analysis of variance (ANOVA) followed by Tukey’s multiple comparisons test was employed. The Kruskal–Wallis test was used for data that did not follow a normal distribution. A *P* value of <0.05 suggested a statistically significant difference.

## RESULTS

### Donors with High Abundance of *Bacteroides* and *Faecalibacterium* Ameliorate DSS-Induced Colitis in Mice

**Figure 1.**
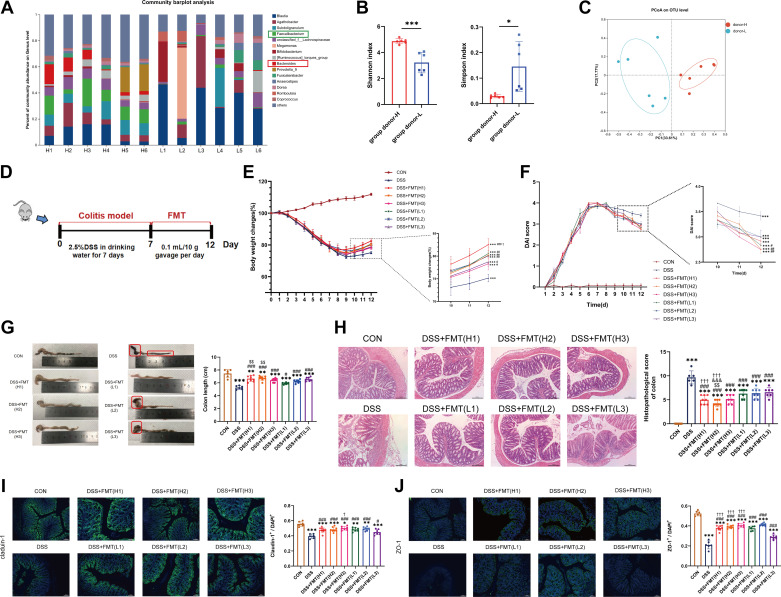
Donors with different bacterial abundance ameliorate colitis differently by FMT. *A*: taxonomic composition distribution on genus-level of donor fecal microbiota in *group donor H* and *group donor L*. *B*: α-diversity (Shannon index and Simpson index) of donor fecal microbiota in *group donor H* and *group donor L*. *C*: principal coordination analysis (PCoA) based on OTU abundance of donor fecal microbiota in *group donor H* and *group donor L*. *D*: mice (*n* = 6–8/group) were administered 2.5% DSS in drinking water for 7 days and then were transplanted daily with fresh feces (0.1 mL/10 g/day) from different donors for 5 days. Daily changes in body weight (*E*) and disease activity index (DAI) (*F*) in different groups. *G*: macroscopic observation of colons. *H*: histopathological changes of colonic samples observed by H&E staining. Photomicrographs of Claudin-1 (*I*) and ZO-1 immunofluorescence (*J*) in the colon (×200 magnification). Data are provided as means ± SE. In *B*, *P < 0.05 and ***P < 0.001. In *E–J*, *means compared with the CON group; #means compared with the DSS group. When comparing between *group H* and *group L*, $means compared with L1; &means compared with L2; † means compared with L3. *, #, and †*P* < 0.05; **, ##, and $$*P* < 0.01; ***, ###, &&&, and †††*P* < 0.001. DSS, dextran sulfate sodium; FMT, fecal microbiota transplantation; H&E, hematoxylin-eosin.

Forty healthy volunteers were screened and divided into *group donor H* and *group donor L* as described previously. We defined the top six donors as H1-6 and the last six donors (donors ranked 40th–35th) as L1-6 (Supplemental Fig. S1). The microbiota composition on genus level for these 12 donors is shown in Fig. 1*A.* Shannon index and Simpson index indicated that the microbial biodiversity of *group donor L* was significantly lower than that of *group donor H* (*P* < 0.05) (Fig. 1*B*). Principal component analysis (PCoA) revealed that the gut microbiota composition of *group donor H* was clearly different from that of *group donor L* (Fig. 1*C*). We selected the top three (H1, H2, and H3) and the last three (L1, L2, and L3) donors for follow-up experiments.

The scheme of inducing colitis in mice and administering FMT therapy is shown in Fig. 1*D.* Compared with the CON group, mice in the DSS group exhibited significant body weight loss and severe intestinal injure (*P* < 0.05) (Fig. 1, *E–J*). After receiving FMT treatment, mice with colitis showed significant improvements in body weight loss, disease activity index (DAI) scores, shortening of colon length, colonic histopathology, and colonic tight junction proteins (TJPs, Claudin-1 and ZO-1) (*P* < 0.05), although there were still differences compared with the CON group (*P* < 0.05) (Fig. 1, *E–J*). In addition, compared with the FMT *group L*, weight loss of mice in FMT *group H* was significantly ameliorated (*P* < 0.05) (Fig. 1*E*). Colon length shortening was more obvious in *group L* than that in *group H* (*P* < 0.05) (Fig. 1*G*). Compared with *group L*, colonic histopathology and TJPs were significantly improved in *group H* (*P* < 0.05) (Fig. 1, *H–J*).

**Figure 2.**
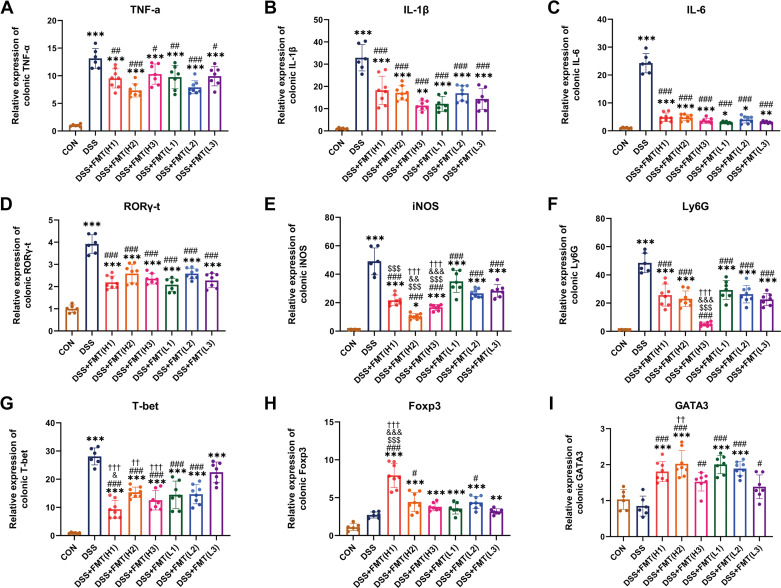
Changes of colonic cytokines and immune cells in colitis with FMT therapy from donors after different bacterial abundance. mRNA expression levels of colonic TNF-α (*A*), IL-1β (*B*), and IL-6 (*C*). mRNA expression levels of colonic RORγ-t (*D*), iNOS (*E*), Ly6G (*F*), T-bet (*G*), Foxp3 (*H*), GATA3 (*I*). Data are provided as means ± SE (*n* = 6–8/group). *Means compared with the CON group; #means compared with the DSS group. When comparing between *group H* and *group L*, $means compared with L1; & means compared with L2; †means compared with L3. *, #, &*P* < 0.05; **, ##, &&, and ††*P* < 0.01; ***, ###, $$$, &&&, and †††*P* < 0.001. DSS, dextran sulfate sodium; FMT, fecal microbiota transplantation; Foxp3, forkhead box protein P3; GATA3, GATA binding protein 3; iNOS, inducible nitric oxide synthase; Ly6G, lymphocyte antigen 6 G; RORγ-t, retinoid-related orphan nuclear receptor γ-t; T-bet, T-box expressed in T cell; TNF-α, tumor necrosis factor-α.

Mice in the DSS group exhibited severer colonic inflammation than the CON group (Fig. 2, *A–C*). Compared with the DSS group, both FMT *group H* and *L* exhibited significantly reduced levels of colonic inflammatory factors [tumor necrosis factor-α (TNF-α), IL-1β, and IL-6] (*P* < 0.05) (Fig. 2, *A–C*). Although no significant difference was found between *group H* and *L*, the inflammatory factors in both groups were significantly higher than the CON group (*P* < 0.05) (Fig. 2, *A–C*). The corresponding changes in colonic immune cells were then observed. Mice in the DSS group showed higher mRNA expression levels of Th17 marker retinoid-related orphan nuclear receptor γ-t (RORγ-t), macrophage marker inducible nitric oxide synthase (iNOS), neutrophil marker lymphocyte antigen 6 G (Ly6G), and Th1 marker T-box expressed in T cell (T-bet) than those in the CON group (*P* < 0.05) (Fig. 2, *D–G*), and there were no differences in the levels of the Treg marker forkhead box protein P3 (Foxp3) and Th2 marker GATA binding protein 3 (GATA3) between the DSS and CON groups (Fig. 2, *H* and *I*). After receiving FMT treatment, mice in both FMT *group L* and *H* exhibited a reduction in RORγ-t, iNOS, Ly6G, and T-bet (*P* < 0.05) (Fig. 2, *D*–*G*) and an increase in Foxp3 and GATA3 compared with the DSS group (Fig. 2, *H* and *I*). In addition, there were no significant differences in ROR γ-t between *group H* and *group L* (Fig. 2*D*). Compared with *group L*, iNOS, Ly6G, and T-bet were decreased in *group H* (*P* < 0.05) (Fig. 2, *E–G*), whereas Foxp3 and GATA3 were increased (*P* < 0.05) (Fig. 2, *H* and *I*). However, the expression levels of all these indicators were still higher than those of the CON group (*P* < 0.05) (Fig. 2, *D–I*). These results suggest that colonic inflammation and immune imbalance were ameliorated after FMT intervention, which was more obvious from donors with high abundance of *Bacteroides* and *Faecalibacterium*.

### Higher Abundance of *Bacteroides thetaiotaomicron* and *Faecalibacterium prausnitzii* in *Group Donor H* than *Group Donor L*

To explore which bacteria in *Bacteroides* and *Faecalibacterium* play the major role in FMT, a combination of relative and absolute quantification RCR was used in these two groups (27). To mitigate the impact of fluctuations in gut microbiota, we regularly collected fecal samples from these six donors every other week for a period of 8 wk, resulting in a collection of 24 samples, 12 samples per group for analysis.

**Figure 3.**
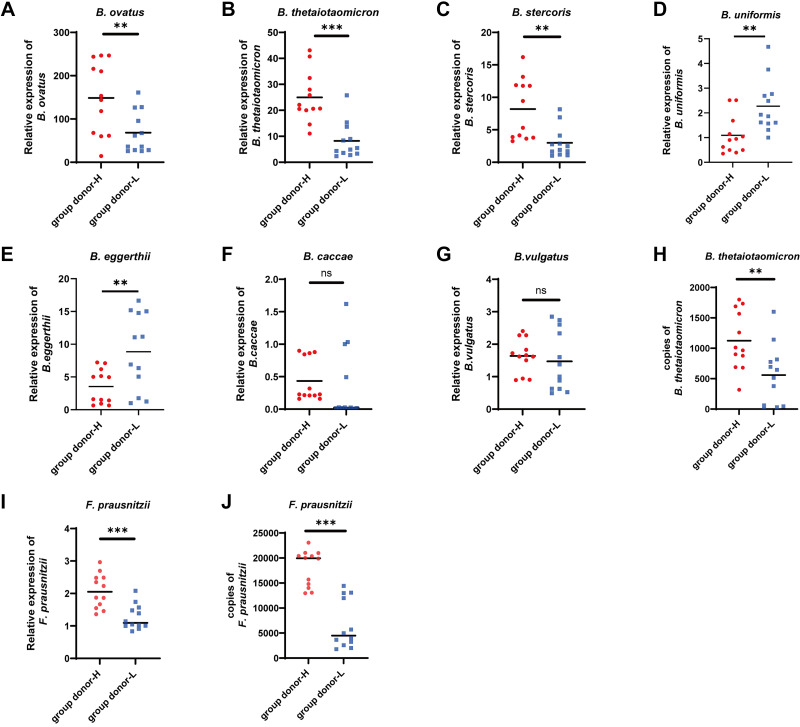
*B. thetaiotaomicron* and *F. prausnitzii* are the key differentiating bacteria in the two groups of fecal microbes. Relative quantitative levels of *B. ovatus* (*A*), *B. thetaiotaomicron* (*B*), *B. stercoris* (*C*), *B. uniformis* (*D*), *B. eggerthii* (*E*), *B. caccae* (*F*), and *B. vulgatus* (*G*) in donor feces. *H*: absolute quantitative levels of *B. thetaiotaomicron* in donor feces by droplet digital PCR (ddPCR). *I*: relative quantitative levels of *F. prausnitzii*. *J*: absolute quantitative levels of *F. prausnitzii* in donor feces by ddPCR. Data are provided as means ± SE (*n* = 12/group). ns, not significant; **P < 0.01 and ***P < 0.001.

The genus *Bacteroides* comprises numerous members, with 40 species having been identified and characterized, among which 24 species are found in the human gut (28). We focused on the most common seven strains that had been reported to possess potential anti-inflammatory effects in IBD (28). *Bacteroides fragilis* was not included in our study due to the documentation of toxin-producing strains (29). We found that *B. ovatus*, *B. thetaiotaomicron*, and *B. stercoris* were statistically higher in *group donor H* than *group donor L* (*P* < 0.05) (Fig. 3, *A–C*). The abundance of *B. uniformis* and *B. eggerthii* in *group donor H* was significantly lower than in *group donor L* (*P* < 0.05) (Fig. 3, *D* and *E*). *B. caccae* and *B. vulgatus* in *group donor H* were lower than *group donor L*, although no significant difference was found (Fig. 3, *F* and *G*). Absolute quantitative analysis showed that *B. thetaiotaomicron* was significantly higher in *group donor H* than in *group donor L* (*P* < 0.05) (Fig. 3*H*). *F. prausnitzii* is the most widely studied and representative member of the *Faecalibacterium* and both real-time PCR and ddPCR showed that *F. prausnitzii* was significantly higher in *group donor H* than in *group donor L* (*P* < 0.05) (Fig. 3, *I* and *J*). Therefore, we focused on *B. thetaiotaomicron* and *F. prausnitzii* in our study.

### Dual-Bacterial Therapy Is Protective against DSS-Induced Colitis

**Figure 4.**
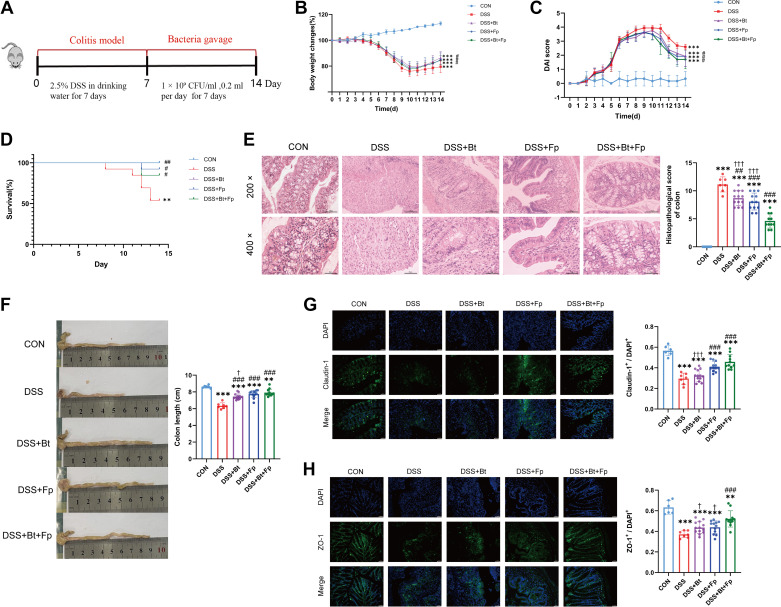
Dual-bacterial therapy is protective against DSS-induced colitis. *A*: schematic of therapeutic administration of bacteria in DSS-induced colitis model. Daily changes in body weight (*B*), disease activity index (DAI) (*C*), and survival in different groups (*D*). *E*: histopathological changes of colonic samples observed by H&E staining and pathological scores of colons. *F*: macroscopic observation of colons and the lengths of colon from each group. *G* and *H*: photomicrographs of claudin-1 (*G*) and ZO-1 immunofluorescence(*H*) in the colon (×200 magnification). Data are provided as means ± SE (*n* = 6–13/group). *Means compared with the CON group; #means compared with the DSS group; †means compared with the DSS+Bt+Fp group. # and †*P* < 0.05; ** and ##*P* < 0.01; ***, ###, and †††*P* < 0.001. DSS, dextran sulfate sodium; H&E, hematoxylin-eosin.

*B. thetaiotaomicron* and *F. prausnitzii* were administered to DSS-induced colitis mice via mono- or dual-bacterial gavage (Fig. 4*A*). Mice in the DSS group exhibited severer weight loss, DAI, mortality rate, and intestinal injure than the CON group (*P* < 0.05) (Fig. 4, *B–H*). Compared with the DSS group, weight loss, DAI, and mortality were significantly ameliorated in all three bacteria-gavage groups (*P* < 0.05) (Fig. 4, *B–D*). There was no significant difference in the aforementioned indicators between dual-bacterial therapy group and mono-bacterial therapy groups but a decreasing trend was observed in dual-bacterial therapy group. Colonic pathology showed that bacteria-gavage therapy alleviated colonic inflammation, mucosal injury, and crypt damage (Fig. 4*E*). Colonic epithelial cellular morphology in dual-bacterial therapy group showed greater improvement than that in both mono-bacterial therapy groups (*P* < 0.05). The colon length shortening was significantly improved in all three bacteria-gavage groups compared with the DSS group (*P* < 0.05) (Fig. 4*F*). The intestinal TJPs claudin-1 and ZO-1 were also significantly restored in all three bacteria-gavage groups, especially in the DSS+Fp group and the DSS+Bt+Fp group (Fig. 4, *G* and *H*). Both mono- and dual-bacterial treatments improved the general and pathological symptoms of colitis in mice, and dual-bacterial therapy exhibited more pronounced efficacy in ameliorating colonic pathological damage, shortened colon length, and TJPs than mono-bacterial therapy (*P* < 0.05) (Fig. 4, *E–G*), although there were still differences compared with the CON group (*P* < 0.05) (Fig. 4, *B–H*).

**Figure 5.**
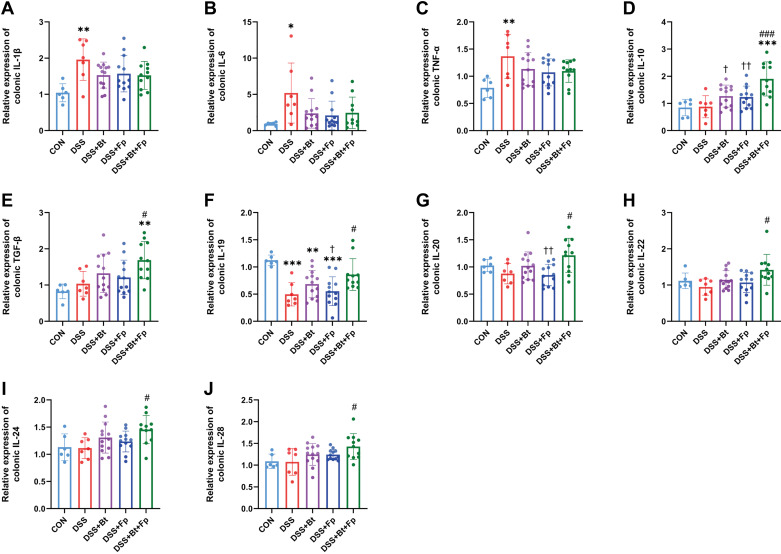
Dual-bacterial therapy modulates the levels of inflammation in colitis. mRNA expression levels of colonic IL-1β (*A*), IL-6 (*B*), TNF-α (*C*), IL-10 (*D*), TGF-β (*E*), IL-19 (*F*), IL-20 (*G*), IL-22 (*H*), IL-24 (*I*), and IL-28 (*J*). Data are provided as means ± SE (*n* = 6–13/group). *Means compared with the CON group; #means compared with the DSS group; †means compared with the DSS+Bt+Fp group. *, #, and †*P* < 0.05; ** and ††*P* < 0.01; *** and ###*P* < 0.001. DSS, dextran sulfate sodium; TNF-α, tumor necrosis factor-α.

The colonic proinflammatory factors including TNF-α, IL-1β, IL-6, and anti-inflammatory factor IL-10 were detected by real-time PCR. Colonic inflammation in the DSS group was significantly severer than that in the CON group (*P* < 0.05) (Fig. 5, *A–J*). Compared with the DSS group, the levels of the proinflammatory factors decreased in all three bacteria-gavage groups without significant difference (Fig. 5, *A–C*). Significantly increased levels of IL-10 were found in dual-bacterial therapy group compared with the DSS group (*P* < 0.05) (Fig. 5*D*), while the increase trend was not significant in mono-bacterial therapy group. Therefore, we further examined classical anti-inflammatory factors such as transforming growth factor-β (TGF-β) and other IL-10 family members as shown in Fig. 5. All the anti-inflammatory factors (TGF-β, IL-19, IL-20, IL-22, IL-24, and IL-28) were significantly increased in dual-bacterial therapy group compared with those in the DSS group (*P* < 0.05) (Fig. 5, *E–J*). There was no significant difference between mono-bacterial therapy groups and the DSS group. Moreover, compared with the CON group, the dual-bacterial therapy group showed a significant increased levels of IL-10 and TGF-β (*P* < 0.05) (Fig. 5, *D* and *E*), indicating anti-inflammatory effects of dual-bacterial therapy.

### Dual-Bacterial Therapy Modulates Intestinal Immune Cells Imbalance

**Figure 6.**
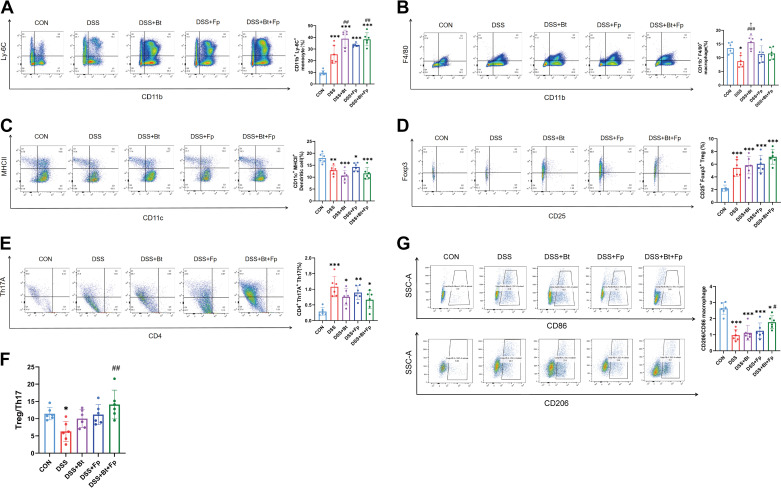
Dual-bacterial therapy modulates immune cells in the lamina propria of the colon. *A*–*G*: flow cytometry analysis of immune cells in the lamina propria of the colon. Monocyte (*A*); macrophage (*B*); dendritic cell (*C*); regulatory T cell (*D*); T helper 17 cell (*E*); ratio of Treg to Th17 (*F*); M2-macrophages and M1-macrophages and their ratios (*G*). Data are provided as means ± SE (*n* = 6/group). *Means compared with the CON group; #means compared with the DSS group; †means compared with the DSS+Bt+Fp group. *, #, and †*P* < 0.05; ** and ##*P* < 0.01; *** and ###*P* < 0.001. DSS, dextran sulfate sodium.

Modulating the host immunity response by gut microbiota may be an effective strategy in attenuating intestinal inflammation, so that the immune cells in gut were assessed. Considering monocytes-macrophages, dendritic cells (d), and Tregs as the main producers of anti-inflammatory factors and playing a crucial role in immune activation and shaping in IBD (30), we detected the changes in the innate immune system [monocytes, macrophages, and dendritic cells (DCs)] and adaptive immune system (Tregs and Th17) by flow cytometry. We found that mice in the DSS group showed lower levels of colonic CD11b^+^F4/80^+^ macrophages, CD11c^+^MHC II^+^ DCs, and higher levels of CD11b^+^Ly-6C^+^ monocytes, Foxp3^+^ Tregs, and Th17 than the CON group (*P* < 0.05) (Fig. 6, *A–F*). Compared with the DSS group, colonic monocyte levels in dual-bacterial therapy group were significantly increased (*P* < 0.05) (Fig. 6*A*), while macrophages tended to increase and DCs tended to decrease with no significant difference (Fig. 6, *B* and *C*). Meanwhile, a rising trend of Tregs and a decreasing trend of Th17 were found in dual-bacterial therapy group (Fig. 6, *D* and *E*), presenting a significant increase in Treg/Th17 ratio (*P* < 0.05) (Fig. 6*F*). The classical proinflammatory morphology-M1 type (CD86^+^) and the anti-inflammatory morphology-M2 type (CD206^+^) of macrophages were detected, and the macrophage M2/M1 ratio increased significantly (*P* < 0.05) after dual-bacterial treatment (Fig. 6*G*). However, there were still differences compared with the CON group (*P* < 0.05) (Fig. 6, *A–G*). These data closely support the hypothesis that dual-bacterial therapy promoted intestinal immune cells toward anti-inflammatory development.

**Figure 7.**
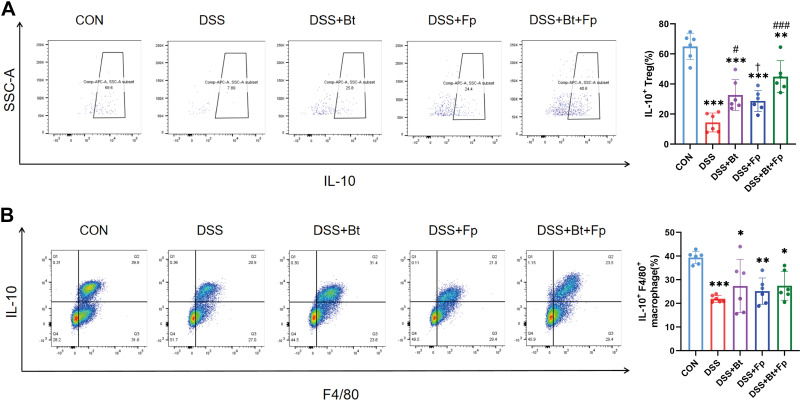
Dual-bacterial therapy promotes the release of IL-10 from Treg. *A* and *B*: flow cytometry analysis of immune cells in the lamina propria of the colon. IL-10 secreting Treg (*A*); IL-10 secreting macrophages (*B*). Data are provided as means ± SE (*n* = 6/group). *Means compared with the CON group; #means compared with the DSS group; †means compared with the DSS+Bt+Fp group. *, #, and †*P* < 0.05; **P < 0.01; *** and ###*P* < 0.001. DSS, dextran sulfate sodium.

Since the intestinal IL-10 is mainly produced by monocyte-macrophages and Tregs, we examined the changes in the ratio of IL-10-producing macrophages and IL-10-producing Tregs by flow cytometry. We found that mice in the DSS group showed a significant decrease in IL-10-producing Tregs and macrophages compared with the CON group (*P* < 0.05) (Fig. 7, *A* and *B*). Moreover, IL-10-producing Treg cells were significantly elevated in dual-bacterial therapy group (*P* < 0.05) (Fig. 7*A*), whereas IL-10-producing macrophages only showed a slight elevation compared with the DSS group (*P* > 0.05) (Fig. 7*B*). The results indicated that dual-bacterial therapy may be mainly conducted by promoting IL-10 production in Tregs.

### Metabolites in Dual-Bacterial Therapy Group Relieve Colitis

Metabolites of gut microbiota are crucial mediators of the interaction between gut microbiota and host (31). We took the contents of the cecum for metabolomic analysis to find out the key metabolites in the dual-bacterial treatment to affect the immune system and alleviate colitis.

**Figure 8.**
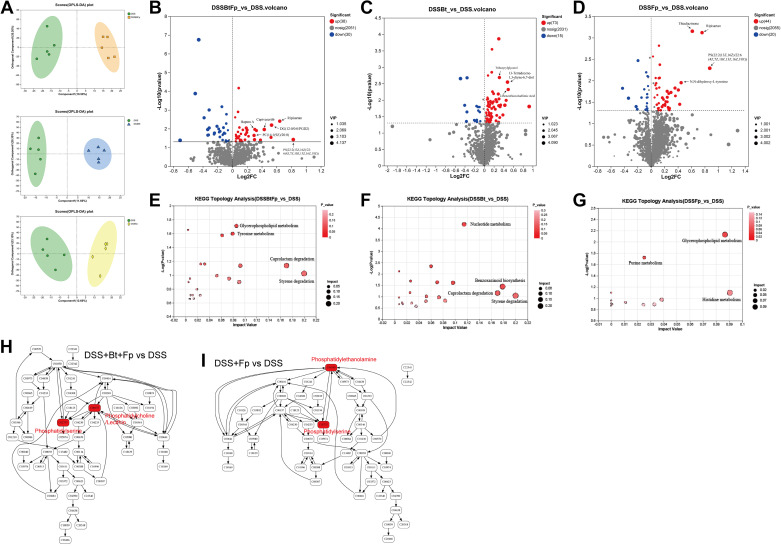
Metabolomic data profiles and pathway enrichment analysis. *A*: orthogonal partial least squares-discrimination analysis (OPLS-DA) for DSS+Bt+Fp vs. DSS, DSS+Bt vs. DSS, DSS+Fp vs. DSS. Volcano plot of changed metabolites for DSS+Bt+Fp vs. DSS (*B*), DSS+Bt vs. DSS (*C*), DSS+Fp vs. DSS (*D*). Kegg pathway enrichment analysis of changed metabolites for DSS+Bt+Fp vs. DSS (*E*), DSS+Bt vs. DSS (*F*), DSS+Fp vs. DSS (*G*). Altered metabolites in glycerophospholipid metabolic pathways in DSS+Bt+Fp vs. DSS (*H*) and DSS+Fp vs. DSS (*I*). Data are provided as means ± SE (*n* = 5/group). DSS, dextran sulfate sodium.

Orthogonal partial least squares-discriminant analysis (OPLS-DA) analysis revealed significant differences in the metabolite composition of the DSS+Bt+Fp group, the DSS + Bt group, and the DSS+Fp group versus the DSS group, demonstrating that both mono- and dual-bacterial treatments significantly altered the metabolite composition of the gut microbiota (Fig. 8*A*). Combining Variable Importance in Projection (VIP) values, *t* test, and fold change (FC)-test analyses, we found that diacylglycerol (12:0/0:0/PGD2) (DG), capivasertib, ripisartan, and hapten A were significantly elevated in the DSS+Bt+Fp group compared with the DSS group (Fig. 8*B*). Compared with the DSS group, 2-benzothiazolsulfonic acid, 13-tetradecene-1,3-diyne-6,7-diol, and tributyrylglycerol significantly elevated in the DSS+Bt group (Fig. 8*C*). Compared with the DSS group, the metabolites that significantly elevated in the DSS+Fp group were phosphatidylserine [22:2(13Z,16Z)/22:6(4Z,7Z,10Z,13Z,16Z,19Z)] (PS), thiadiazinone, ripisartan, and *N*,*N*-dihydroxy-l-tyrosine (Fig. 8*D*). Of these, DG, the metabolite with the highest VIP ranking in the dual-bacterial treatment group, is a member of the diacylglycerol family and an important substrate for the biosynthesis of triglycerides or phosphatidylcholine (PC, lecithin), phosphatidylserine (PS), and phosphatidylethanolamine (PE). Its downstream lecithin and PS were significantly increased in the DSS+Bt+Fp group, whereas PS and PE were significantly increased in the DSS+Fp group (Fig. 8, *B* and *D*). This indicated that the most significantly changed metabolites after bacterial treatment were mainly related to glycerophospholipid metabolism. Moreover, the Kyoto Encyclopedia of Genes and Genomes (KEGG) pathway enrichment analysis revealed that compared with the DSS group, the glycerophospholipid metabolism pathway was significantly upregulated in the DSS+Bt+Fp group and the DSS+Fp group, which in accordance with the previous analysis of the difference in content level (Fig. 8, *E–G*). KEGG pathway enrichment analysis revealed that in the glycerophospholipid pathway, lecithin and PS were upregulated in DSS+Bt+Fp versus DSS groups, whereas PE and PS were upregulated in DSS+Fp versus DSS groups (Fig. 8, *H* and *I*). Lecithin was identified as a crucial product in the comparison of DSS+Bt+Fp versus DSS+Fp groups. Our analysis revealed that there were no substances related to glycerophospholipid metabolism among the products that were significantly elevated in the DSS+Bt group. We hypothesized that *B. thetaiotaomicron* may contain genes encoding relevant enzymes, and it is necessary for *F. prausnitzii* to provide substrates to facilitate the transformation of PE into lecithin. In conclusion, metabolite analysis revealed that dual-bacterial intervention significantly increased metabolites related to glycerophospholipid metabolism, especially lecithin, thereby modulating intestinal immunity and ameliorating colitis.

## DISCUSSION

The incidence and prevalence of IBD are increasing globally every year. FMT, as a treatment for IBD, works primarily by modifying the colonic ecosystem. The microbiota dysbiosis, including decreased microbial diversity, reduced beneficial microbiota, and increased opportunistic pathogens, has been recognized as an important contributing factor in UC pathogenesis (32). Disturbance of the microbial-derived metabolites such as short-chain fatty acids (SCFAs), secondary bile acids (SBA), and tryptophan metabolites has also been observed in patients with UC (33). Although the efficiency of FMT in patients with UC remains controversial (34–37), it partially restores microbial changes microbial and metabolomics profiles in responders toward donor profiles (38). In addition, the impaired intestinal barrier function and intestinal immune regulation in UC lead to persistent inflammation mainly manifested by the dysregulation of immune balance (39, 40). The immune response of FMT in UC mainly focused on T cells and revealed divergent effects on pro-/anti-inflammatory functions (38). Our previous study also found that the modification of serum TNF-α, IL-6, and IL-10 might be related to the efficiency of FMT in UC (41).

The efficiency of FMT therapy is likely influenced by factors such as the dosage, frequency of treatment, and especially the microbial composition of donors and recipients (34). Therefore, we searched the impact of different donors on FMT efficacy and focused on targeted bacteria and their potential influence on host immune response.

Literature reported that the significant decreased abundance of *Bacteroides* and *Faecalibacterium* was associated with disease activity and progression in patients with IBD (6, 32). We screened donors based on the abundance of these two genera by sequencing results of 16S rRNA and then performed FMT therapy for colitis in mice. Donors’ feces with high abundance of *Bacteroides* and *Faecalibacterium* were found to be more effective in ameliorating colitis, which confirmed the influence of different microbial compositions in donors on treatment efficacy. To further investigate the potential mechanisms, certain species were focused on. In this study, we used a combination of real-time PCR and digital PCR to detect the feces of donors over a period in search of the species with the most significant differences which were found to be *B. thetaiotaomicron* and *F. prausnitzii*. The therapeutic effects of these two individual bacterial strains have been explored in IBD. The bacterial sphingolipids of *B. thetaiotaomicron* are crucial for maintaining intestinal homeostasis (42). Li et al. (9) found that *B. thetaiotaomicron* can activate the AHR pathway through tryptophan-related metabolites, thereby regulating the balance of Th17/Treg and alleviating colitis. *F. prausnitzii* can regulate Treg/Th17 by inhibiting HDAC 1 and 3 through the metabolite butyrate (12, 13). *F. prausnitzii* can ameliorate colitis by inhibiting the NF-κB pathway to reduce the production of IL-8 through secretion of microbial anti-inflammatory molecule (MAM) (43, 44). However, the study including both bacteria in colitis has not been fully investigated.

The interaction between microbiota plays a critical role in the pathogenesis of IBD (45). In previous study, Murakami et al. (46) cocultured some *Bacteroides* strains with *Faecalibacterium prausnitzii* in vitro and found that *Bacteroides* strains could promote the growth of *F. prausnitzii*, based on the fact that *Bacteroides* strains could metabolize polysaccharides to produce acetate, whereas *F. prausnitzii* could metabolize acetate to butyrate. It is speculated that their synergistic effect may be beneficial in the maintenance of homeostasis (46, 47). Therefore, we conducted our research on the combination therapy of dual-bacteria for colitis and found that dual-bacterial therapy showed better effects in regulating colonic inflammation and immune imbalance than mono-bacterial therapy.

Patients with IBD exhibit intestinal immune imbalance, primarily manifested as alterations in the ratio of Treg to Th17 cells (38, 48). Besides, in mice with colitis, there is a decrease in Tregs and an increase in Th17 cells in the peripheral blood (49, 50). In addition to adaptive immunity, innate immunity also plays a crucial role in initiating and determining local and systemic immune responses in IBD (30). In our experiment, after dual-bacterial therapy, the level of monocytes significantly increased, while the levels of macrophages and dendritic cells exhibited no significant changes. In addition, the ratios of M2/M1 and Treg/Th17 and IL-10-producing Tregs significantly increased after dual-bacterial therapy, indicating a shift in intestinal immunity from proinflammatory to anti-inflammatory state. The anti-inflammatory cytokine IL-10 has been linked to the etiology of IBD through multiple genome-wide association studies. Spontaneous IBD occurs in both mice and human when there are genetic disturbances in IL-10 or its receptor IL-10R (51). Although it has reported that IL-10 produced by macrophages is associated with the development of IBD (51), Zigmond et al. (52) found that it is not specific depletion of IL-10, but specific deficiency of IL-10R in intestinal macrophages, affecting intestinal homeostasis and the differentiation of Tregs. Unlike macrophage, specific deletion of IL-10 in CD4^+^ T cells (∼80% are Tregs) leads to the development of spontaneous colitis in mice, similar to the phenotype of complete IL10^−/−^ mice (53, 54). These findings suggested that IL-10-producing Tregs, not macrophages, are likely the determining factor in the progression of intestinal inflammation during colitis. In our experiments, the anti-inflammatory effects mainly rely on the increase in IL-10-producing Tregs after dual-bacterial therapy, consistent with previous literature.

Beneficial bacteria can regulate intestinal immune homeostasis and alleviate inflammation, which might be mediated by metabolites (39, 55). In our study, we found that the differential substances were mainly associated with glycerophospholipid metabolism, which was consistent with the KEGG enrichment pathway analysis. It was observed that the levels of lecithin were elevated in dual-bacterial therapy group, which was not significantly changed after mono-bacterial therapy. Literature reported cross-feeding effect to promote *F. prausnitzii* growth and its metabolite when cocultured with *Bacteroides* strains (46). Therefore, we speculated that specific enhanced metabolites in dual-bacterial therapy such as lecithin can regulate immune imbalance. Lecithin is a kind of lipid, which significant changes in the lipid profile of patients with IBD (56). Soh et al. (57) found that the levels of serum triglyceride decreased in patients with UC may be associated with the degree of inflammation. In addition, there were significant changes in lipids related to glycerophospholipid, linoleic acid, and sphingomyelin metabolism in patients with IBD (58). Alterations in lipid profile may occur before clinical diagnosis, in the subclinical stages, or early phases of disease (57). Therefore, monitoring lipid changes as a risk factor for IBD might aid in early diagnosis and treatment of disease. Studies have found that supplementing with lipid such as lecithin can improve symptoms of IBD (59). Animal experiments also confirmed that lecithin has a relieving effect on DSS-induced colitis (60). Integrated analysis of metabolomics and microbiomics indicated that lecithin supplementation can regulate endogenous tryptophan metabolism, arginine and proline metabolism, purine metabolism, bile secretion, and vitamin digestion, and absorption by regulating gut microbiota (61). However, the impact of lecithin on Tregs has not yet been validated and this needs to be further investigated in our future work.

In summary, our research focuses on the key gut microbiota in FMT therapy for IBD. We propose the key microbial communities of effective FMT and briefly elucidate the functional mechanisms of their role in terms of immunity and metabolites.

## DATA AVAILABILITY

The raw data for 16S rRNA sequencing of this study are openly available in BioProject with reference number PRJNA1070265.

## SUPPLEMENTAL DATA

10.6084/m9.figshare.24894396.v4 Supplemental Figs. S1 and S2: https://doi.org/10.6084/m9.figshare.24894396.v4.

10.6084/m9.figshare.24894432.v1 Supplemental Table S1: https://doi.org/10.6084/m9.figshare.24894432.v1.

## GRANTS

This study was funded by the National Natural Science Foundation of China (No. 81970555, 82270671), Songjiang Science and Technology Committee (22SJKGGG28), and Shanghai Jiao Tong University School of Medicine, Digestive Institute (KY-2023-03-02).

## DISCLOSURES

No conflicts of interest, financial or otherwise, are declared by the authors.

## AUTHOR CONTRIBUTIONS

B.X., Y.Z., and C.H. conceived and designed research; B.X., Y.F., N.Y., and W.Q. performed experiments; Z.H. and W.X. analyzed data; H.H., Q.M., and J.F. interpreted results of experiments; B.X., Z.H., and W.X. prepared figures; B.X. drafted manuscript; Y.Z. and C.H. edited and revised manuscript; B.X., Y.F., N.Y., W.Q., Z.H., W.X., H.H., Q.M., J.F., Y.Z., and C.H. approved final version of manuscript.
